# On the Reaction Mechanism of the 3,4-Dimethoxybenzaldehyde Formation from 1-(3′,4′-Dimethoxyphenyl)Propene

**DOI:** 10.3390/molecules23020412

**Published:** 2018-02-14

**Authors:** Sebastián Cuesta, Josefa Arias, Felipe Gallegos, Jans Alzate-Morales, Lorena Meneses

**Affiliations:** 1 Chemistry Department, Pontifical Catholic University of Equator, Av. 12 de Octubre 1076 y Roca, Quito 170109, Ecuador; sebas_c89@hotmail.com (S.C.); earias570@puce.edu.ec (J.A.); fgallegos492@puce.edu.ec (F.G.); 2 Center for Bioinformatics and Molecular Simulations, Faculty of Engineering, University of Talca, 2 Norte 685, Casilla 721, Talca 3640000, Chile

**Keywords:** lignin peroxidase, DFT, reaction force, force constant, reaction mechanism, 3,4-dimethoxybenzaldehyde, 1-(3′,4′-dimethoxyphenyl) propene

## Abstract

Lignin peroxidase (LiP) is an important enzyme for degrading aromatic hydrocarbons not only in nature but also in industry. In the presence of H_2_O_2_, this enzyme can easily decompose lignin and analogue compounds under mild conditions. In this reaction mechanism, LiP catalyzes the C–C cleavage of a propenyl side chain, being able to produce veratraldehyde (VAD) from 1-(3′,4′-dimethoxyphenyl) propene (DMPP). One of the few and complete proposed mechanisms includes several non-enzymatic reactions. In this study, we performed a computational study to gain insight about the non-enzymatic steps involved in the reaction mechanism of VAD formation from DMPP using LiP as a catalyst. A kinetic characterization of the reaction using the reaction force and the reaction force constant concepts within the density functional theory (DFT) framework is proposed. All theoretical calculations for the reaction pathway were performed using the Minnesota Global Hybrid functional M06-2X and a 6-31++G(d,p) basis set. The complete reaction comprises seven steps (five steps not including LiP as a catalyst), which include radical species formation, bond transformation, water and oxygen addition, atom reordering, and deacetylation. The overall mechanism is an endothermic process with mixed activation energies depending on the four transition states. These results are the first attempt to fully understand the catalytic role of LiP in the degradation of lignin and its aromatic derivative compounds in terms of the electronic structure methods and future hybrid calculation approaches that we have recently been performing.

## 1. Introduction

Lignin is a complex, heterogeneous, three-dimensional aromatic polymer containing random dimethoxylated, monomethoxylated, and nonmethoxylated phenylpropanoid subunits [1,2,3]. Differently from most biological polymers, lignin subunits are linked in a nonlinear way as a consequence of its synthesis reaction mechanism that is mediated by free radicals [3]. The three most abundant lignin monomers are *p*-coumaryl alcohol, coniferyl alcohol, and sinapyl alcohol, which are synthesized in the cytosol [4]. 

This polymer encompasses 20–30% of all woody plants and it can be found in woody and vascular tissues making the cell wall more rigid and hydrophobic [1,2,3]. More in-depth, lignin is part of the secondary cell wall of plants, being within and between the cell walls of tracheids, vessels, and fibers of xylem tissue filling the spaces between the cellulose, hemicellulose, and pectin components [3,4]. Lignin function includes helping transport aqueous nutrients from the root by providing a hydrophobic capillary surface, protecting the plant from pathogens and microbial attack, and giving the plant extra strength for structural stability [1].

In nature, *white-rot* fungi are the only known organisms able to entirely break down lignin in wood, producing carbon dioxide and water [3,4]. Under the right conditions, these fungi secrete enzymes that catalyze the oxidative depolymerization of lignin, which include heme peroxidases such as lignin peroxidase, manganese peroxidase, and their isozymes [5]. Those enzymes, in the presence of hydrogen peroxide (H_2_O_2_) are essential components of the lignin degradative system [6,7,8]. Lignin peroxidase (LiP) is an extracellular heme protein produced by fungi under nutrient-limiting conditions [7]. This enzyme was the first one to be discovered and isolated from *Phanerochaete chrysosporium* Burds. [1,3,9]. The glycosylated enzymes are composed of around 340 aminoacids with a molecular weight of about 38–50 kDa. As part of their tertiary structure, they contain also a single heme group and two calcium ions [1].

LiP is important for degrading aromatic hydrocarbons not only in nature but also in industry [5]. They have potential applications in a large number of fields, including the paper, cosmetic, food, textile, fuel, chemical, and agricultural industries [6]. LiP can easily decompose lignin and its analogues under mild conditions, lowering the energy input, reducing the environmental impact, and providing a more specific and effective alternative [1,5,9]. It has been suggested that these enzymes degrade lignin and some organopollutants, including indane, pentachlorophenol, polychlorinated biphenyls, pyrene, and cyanide [10]. Most important industry uses for lignin are biopulping, biobleaching, the removal of recalcitrant organic pollutants, and the production of liquid fuels and biochemicals [4,6].

Lignin biodegradation by *P. chrysosporium* has been widely studied [11]. This degradation is an H_2_O_2_-dependent process similar to the one used by other peroxidases [1,11]. Several studies have been conducted in attempts to obtain a better understanding of the complex process of lignin biodegradation, and these studies have consisted in characterizing the enzyme in terms of their structure and biophysical and chemical properties [11]. Other studies have been directed towards elucidating the exact chemical mechanisms involved in lignin sidechain cleavage and aromatic ring-opening reactions [12]. It is a highly accepted view that the reaction mechanism of LiP consists in the one-electron oxidation of aromatic hydrocarbons, catalyzing the cleavage of the C–C bond and splitting off β-*O*-4 linkages [9,13,14].

In this reaction, one of the oxidation products of 1-(3′,4′-dimethoxyphenyl) propene (DMPP) is veratraldehyde (VAD), demonstrating that LiP catalyzes the C–C cleavage reaction of the propenyl side chain [14]. In the mechanism, a veratryl alcohol cation radical is formed as an intermediate [13]. Apart from the LiP-catalyzed one-electron oxidation reaction, this mechanism goes through several non-enzymatic reactions, including water addition and the splitting of a proton to form a radical. Radicals are reactive species that enable side reactions, such as the addition of a superoxide, radical coupling, or peroxyl-radical formation [14].

**Figure 1:**
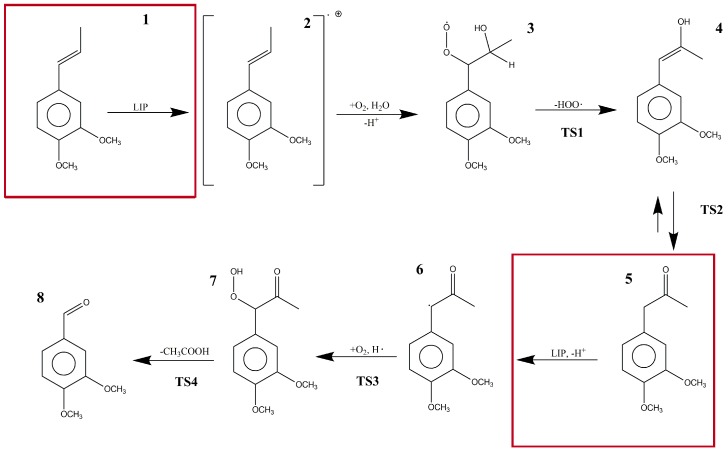
The proposed reaction steps in the bio-catalytic pathway of lignin peroxidase for the formation of veratraldehyde (VAD) from 1-(3′,4′-dimethoxyphenyl) propene (DMPP).

To the best of our knowledge, there have been no previous computational studies that have reported the above-mentioned reaction mechanism at a quantum electronic structure level. Thereby, in this study, we obtained new insights about the different steps involved in the LiP reaction mechanism (Figure 1) using DFT to characterize the reactants, intermediates, transition states, and products proposed by Ten Have et al. [14]. Due to the complexity of the biological model, the steps where LiP participated as a catalyst were not calculated nor described in the main pathway. Additionally, we performed a kinetic characterization of the mechanism using the reaction force and the reaction force constant concepts within the Density Functional Theory (DFT) framework. Red squares in Figure 1 represent the reaction steps in which LiP is supposed to be involved.

## 2. Results

In the studied reaction mechanism, there were several chemical processes such as radical species formation, bond transformation, water and oxygen addition, atom reordering, and deacetylation to form VAD from DMPP. In our solvation phase molecular models, the reaction started in an energetic minimum with reagents in the basal state. LiP interacted with DMPP (**1**) forming DMPP as a cation radical species (**2**). This compound, in the presence of water and molecular oxygen, formed 1-(3,4-dimethoxyphenyl)-1-hydroperoxypropan-2-ol (**3**). In turn, compound **3** experienced an elimination of the peroxyl radical in an endothermic reaction forming (*E*)-1-(3,4-dimethoxyphenyl)-1-propen-2-ol (**4**). Then, an intrinsic proton transfer converted the alcohol into a ketone (keto-enol equilibrium) named 1-(3,4-dimethoxyphenyl)propan-2-one (**5**). At this point, LiP intervened in the reaction taking out a proton forming again a free radical species (**6**). Once more, molecular oxygen took part in the reaction and, with a hydrogen free radical, formed 1-(3,4-dimethoxyphenyl)-1-hydroperoxypropan-2-one (**7**). This hydrogen free radical was formed by the decomposition of the peroxyl radical into O_2_ and H•. Finally, deacetylation of compound **7** occurred, and VAD was formed as the final reaction product (**8**). 

**Figure 2:**
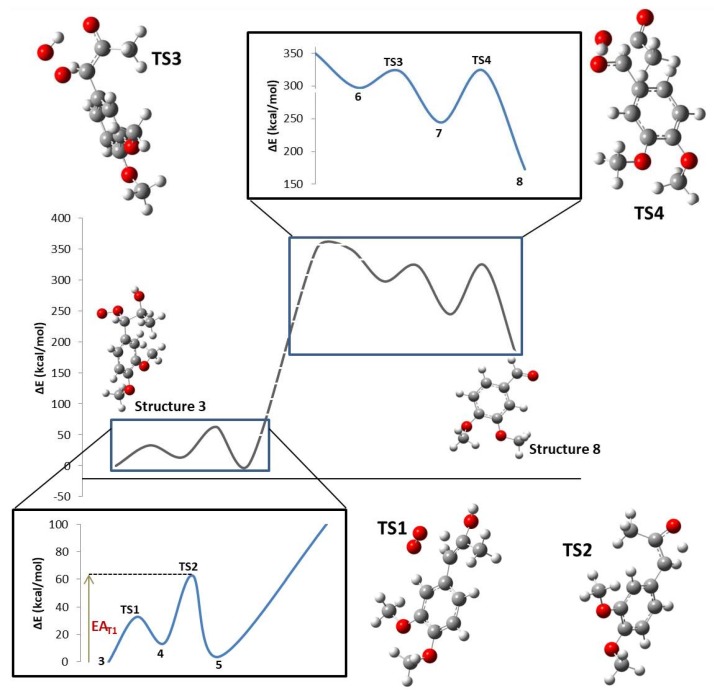
Whole intrinsic reaction coordinate (IRC) for the formation of VAD from DMPP.

At each reaction step, as the reagents (R) became closer to each other and began to interact, steric and electrostatic forces began to change the geometry, causing the potential energy of the system to rise to a maximum energy species, termed the transition state (TS). At this point, bonds, angles, and dihedrals changed drastically. Electronic changes allowed the reactant molecules to form and break bonds between them to finally relax the system to a local energy minimum, termed the product (P). The whole energy profile of the studied reaction is shown in Figure 2, where Molecule **3** was selected as the reference system (ΔE = 0 kcal/mol). The energy profile goes from compound **3** to the end of the main pathway described by compound **8** (VAD formation). The transition state for the transformation of DMPP radical into compound **3** could not be characterized. The reaction enthalpy (172.92 kcal/mol) was calculated by introducing compounds **3** and **8**, hydrogen and acetic acid enthalpies, into Equation (1). A global activation energy could not be found because the mechanism is not complete. In Figure 2, dotted lines represent the reaction step where LiP is involved, so reaction and activation energies were not obtained.

Activation and reaction standard molar energies, enthalpies, entropies, Gibbs free energies, and the reaction work are presented in Table 1. Each thermodynamic value includes its respective electronic, vibrational, rotational, and translational component. Activation energy values went from 25.12 to 79.52 kcal/mol. Reaction enthalpies demonstrated that Step 1 is an endothermic process, while Steps 2–4 are exothermic.

Total reaction and activation energies obtained are similar to the electronic energies, indicating the small contribution that the vibrational, translational, and rotational components make. Reaction Gibbs energy shows that TS2, TS3, and TS4 will occur spontaneously, while TS1 apparently will not. To make an endergonic reaction happen, it can be pulled or pushed by an exergonic process (details see supplementary materials). In this case, it can be pulled from the TS2 reaction (ΔG^0^ = −10.01 kcal/mol) or, only if it is strongly exergonic, it can be pushed from the previous reaction. Gibbs activation energies are all positive, indicating that the reagents will not reach the transition state unless they absorb energy. For entropy, negative S^A^ shows that the reagent reaching the transition state is not a favorable process. Reaction entropies show that only TS3 is not entropically favored. It can be seen that there is no important contribution of the work to the process of activation because, in almost all of the cases, these values are, or near, zero.

**Figure 3:**
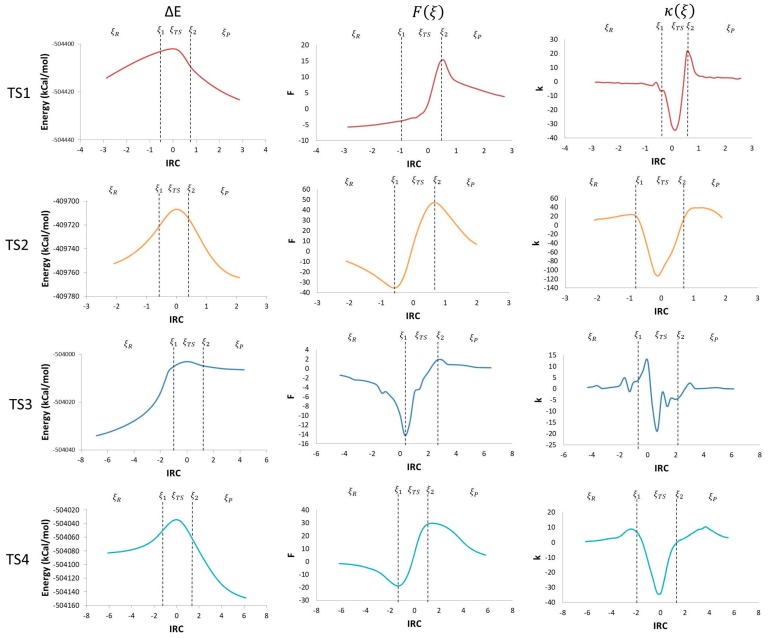
IRC vs. potential energy (kcal/mol), ΔE, reaction force, *F*(*ξ*), and reaction force constant, *k*(*ξ*).

Using the potential energy profile and Equations (3) and (4), which define the reaction force and reaction force constant, the corresponding comparison graphs were plotted along the intrinsic reaction coordinate (IRC) for each TS found in the studied mechanism (Figure 3).

## 3. Discussion

In the mechanism proposed by Ten Have et al. [14], the reaction consists of seven steps from which Steps 1 and 5 involve LiP as a catalyst. Those two reaction steps were not studied in our research work because energies and information obtained from those steps, due to the catalytic effect of the enzyme, would not be accurate enough to be taken into account due to the simplicity of our molecular models. In order to study the catalytic effect of the enzyme in this reaction, we are implementing molecular dynamics and hybrid calculations (i.e., Quantum Mechanics/Molecular Mechanics QM/MM) protocols to obtain a better model of the overall enzymatic catalyzed reaction path. 

The overall studied mechanism is an endothermic process with a mixed activation energy depending on the four transition states located in the present study. This means the system absorbed 172.92 kcal/mol in order to produce VAD from DMPP. This amount of energy is absorbed in the form of heat. Comparing the different steps along the reaction, it was noticeable that one of them is endothermic (TS1) and the other three are exothermic (TS2, TS3, and TS4). This result is in agreement with experimental data where incubation and 30 °C [14] were needed to make the reaction proceed from the reagents to products.

In the modeled reaction mechanism, the step with the highest activation energy in the first part of the mechanism was the proton transfer described in TS2 (49.03 kcal/mol), suggesting that this step could be the rate-determining reaction step of the first part of the reaction. This was somehow expected because of the amount of energy required to break a C–C double bond and to create a C–O double bond. In addition, it is the only step during the entire reaction that does not involve a free radical. In a non-enzymatic reaction, it is expected that the activation energy in the steps involving the formation of free radicals should be higher due to the energy needed to form a more activated molecule with unpaired valence electrons. However, as chemical species having free radicals are more reactive, it can be seen that the reaction potential energy of these different species during the described reaction mechanism is lower than that where neutral chemical species (chemical structures that does not involve a free radical) are participating. The rate-determining step in the second part of the mechanism was TS4 with an activation energy of 79.52 kcal/mol. This step, like TS2, does not involve free radical species. Additionally, it contains a series of high energetic transformations such as bond cleavage, bond formation, and bond transformation.

Taking the derivative of the potential energy along the reaction coordinate, the reaction force was obtained. The reaction force is a quantum descriptor that gives important information about the mechanism of a chemical reaction. In a reaction force plot analysis, three regions are noticeable; the reagents region (*ξ_R_* ≤ *ξ* ≤ *ξ*_1_), the transition state region (*ξ*_1_ ≤ *ξ* ≤ *ξ*_2_), and the products region (*ξ*_2_ ≤ *ξ* ≤ *ξ_P_*), where *ξ_R_* and *ξ_P_* represent the energies of reagents and products, respectively. Those three regions are defined by two critical points: a minimum (*ξ*_1_) and a maximum (*ξ*_2_) (Figure 3). The reaction force values go down to a minimum in the reagent region, showing that structural changes take place in the reaction. In this region, before TS1, reagents get together by reducing the O–C and the H–C distances; before TS2, geometry changes in the O–H and C–H bonds angles, and C–O and C–C bond distance occur; before TS3, the elongation of the O–O bond in the peroxyl radical and a reduction in the C–O distance take place; before TS4, a reduction in angles and an elongation of the C–C bond are the main changes. 

**Figure 4:**
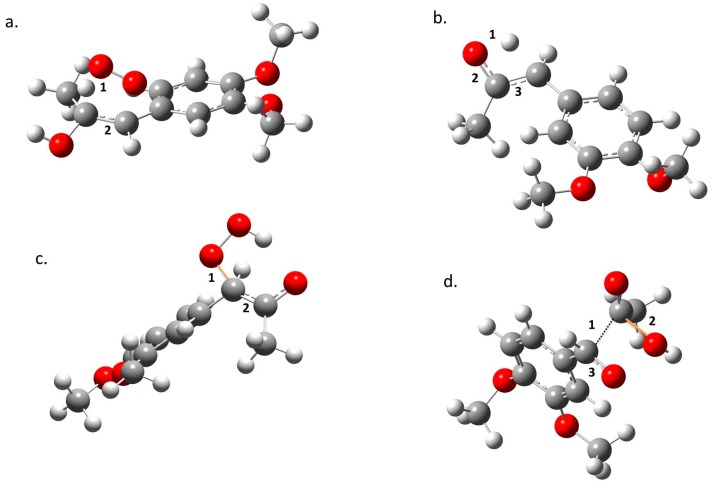
Structural changes taking place in (**a**) TS1, (**b**) TS2, (**c**) TS3, and (**d**) TS4. Oxygen is represented in red, carbon in grey, and hydrogen in white. Forming bonds are presented in orange and numbers show parts of the molecules where changes occur (see text).

In the transition state region, electronic reordering might take place. (Figure 4). Once the geometry adjusts to an optimal position to allow electronic changes to occur, force constant values go up rapidly to a maximum, indicating that these changes are occurring. In TS1, the O–H bond in the peroxyl radical breaks (a1), and the C–C bond goes from a double to a simple bond (a2); in TS2, the O–H bond of the alcohol disappears (b1), the double bond of the ketone forms (b2), and the C–C double bond transforms to a single bond (b3); in TS3, the O–C bond is formed (c1), and the C–C double bond becomes a single bond (c2); in TS4, there is a cleavage of the C–C bond (d1), the C–O bond forms in the acetic acid (d2), and there is a change from a single to a double bond in the C–O bond of the aldehyde (d3). Finally, once all of the electronic changes are completed, the geometry changes again relaxing the system into the most stable form of the product, which translates to a decrease of the force constant values along the reaction force plot [15,16,17,18,19,20,21,22]. 

The negative derivative of the reaction force with respect to the IRC brings us another quantum descriptor: the force constant (*κ*(*ξ*)). Values of the maximum and the minimum obtained from the reaction force profile are represented in Figure 3, as the inflexion points in *ξ*_1_ and *ξ*_2_ are zero. The reaction constant values drop down to negative values during the transition state region, that coincides with values between the reaction force maximum and minimum (*ξ*_1_ → *ξ*_TS_ → *ξ*_2_). During the reagent and product regions, the values remain positive (*ξ_R_* → *ξ*_1_ and *ξ*_2_ → *ξ_P_*) [21]. This shows that there is not a single transition state point in every step of the reaction mechanism, but a transition state region where major structural and electronic changes occurs going from a state of activated reagents to a state of activated products. This translates in force constant values going from a minimum to a maximum [23]. Additionally, the transition state regions have an energy gradient of zero for all the degrees of freedom with a negative reaction force constant [21].

## 4. Materials and Methods 

All computational calculations along the reaction pathway, for the formation of VAD from DMPP, were performed using the Minnesota Global Hybrid functional M06-2X [24] in conjunction with a 6-31++G(d,p) basis set implemented in the GAUSSIAN 09 package of programs (Gaussian Inc., Wallingford, CT, USA) [25]. The Berny analytical gradient optimization algorithm was used in the optimization process of the transition state and a normal mode vibrational analysis was executed to confirm that the resulting optimized structures correspond to a Transition State (TS) or a minimum in the potential energy surface [26]. For the transition states, the unique imaginary frequency associated with the transition vector indicates that they were a true TS structure. All structures were optimized in solution (water) using the SCRF (Self-consistent reaction field) keyword, and a solvation model density (SMD) [27,28]. Structure optimization and energy and vibrational calculations were performed in the reagents, products, and TS structures to obtain reaction and activation energies, enthalpies, free energies, and entropies using Equations (1) and (2) [29]. An IRC calculation was performed to analyze all the changes along the reaction coordinate. Quantum descriptors (reaction force *F*(*ξ*), and force constants *κ*(*ξ*)) described elsewhere [15,16,17,18,19,20,21,22,30,31] were calculated using Equations (3) and (4). The work done by the vibrational, rotational, and translational contributions was calculated using Equation (5).

(1) $$\Delta E^{o}=\sum E_{products}-\sum E_{reactants}.$$

(2) $$E^{A}=E_{TS}-\sum E_{reactants}.$$

(3) $$F\left(\xi \right)=-\frac{dE}{d\xi }.$$

(4) $$\kappa \left(\xi \right)=-\frac{\partial F\left(\xi \right)}{\partial \xi }.$$

Δ(pV) = ΔH − ΔE.(5)

## 5. Conclusions

The studied reaction mechanism for VAD formation from DMPP comprises seven reaction steps, and the five steps that do not include LiP as a catalyst were the focus of this study. Those reaction steps consisted in radical species formation, bond transformation, water and oxygen addition, atom reordering, and deacetylation. The overall mechanism is an endothermic process with mixed activation energies depending on the transition states found. This preliminary information, though it consists of very simple molecular models and does not involve or affect the protein environment or the explicit water solvation in different stages of the reaction, will be useful for setting up a complete substrate–enzyme molecular model where all effects are taken into account by means of molecular dynamics and/or QM/MM methods. Our group is continuing to develop this research and compare activation energies with respect to other LiP substrates.

## Figures and Tables

**Table 1:** Reaction and activation energies, enthalpies, entropies, Gibbs free energies, and reaction work for different reaction steps involved in VAD formation.

Step	TS1	TS2	TS3	TS4
ΔE^0^ (kcal/mol)	13.81	−9.56	−53.17	−71.50
E^A^ (kcal/mol)	32.80	49.03	25.71	79.52
ΔH^0^ (kcal/mol)	14.40	−9.56	−53.76	−70.91
H^A^ (kcal/mol)	32.80	49.03	25.12	79.52
ΔG^0^ (kcal/mol)	2.15	−10.01	−40.58	−83.06
G^A^ (kcal/mol)	32.92	49.36	38.37	81.20
ΔS^0^ (cal/mol.K)	41.10	1.54	−44.21	40.73
S^A^ (cal/mol.K)	−0.42	−1.10	−44.45	−5.63
ΔPV^0^ (kcal/mol)	0.59	0.00	−0.59	0.59
PV^A^ (kcal/mol)	0.00	0.00	−0.59	0.00

ΔE^0^ = Standard molar reaction energy; E^A^ = Standard molar activation energy; ΔH^0^ = Standard molar reaction enthalpy; H^A^ = Standard molar activation enthalpy; ΔG^0^ = Standard molar Gibbs free energy; G^A^ = Standard molar Gibbs free activation energy; ΔS^0^ = Standard molar entropy; S^A^ = Standard molar activation entropy; ΔPV^0^ = Reaction work; PV^A^ = Activation work.

## Supplementary Materials

Supplementary materials are available online.
