# Supplementary material for: On the Reaction Mechanism of the 3,4-Dimethoxybenzaldehyde Formation from 1-(3′,4′-Dimethoxyphenyl)Propene
Source: Molecules. 2018 Feb 14;23(2):412. doi: 10.3390/molecules23020412 (PMC6017041; doi:10.3390/molecules23020412)
Supplement: Supplementary file 1 [file molecules-23-00412-s001.zip › Supplementary material 1..pdf]

Table S1: Total reaction and activation energies, enthalpies, entropies and Gibbs free energies

Structure TS1	Hartrees	kcal/mol
Energy	-803.5522	-504237
Enthalpy	-803.5513	-504236.5
Gibbs	-803.6143	-504276
Entropy	132.652	cal/mol.K

Structure TS2	Hartrees	kcal/mol
Energy	-652.6689	-409556.3
Enthalpy	-652.668	-409555.7
Gibbs	-652.7242	-409591
Entropy	118.476	cal/mol.K

Structure TS3	Hartrees	kcal/mol
Energy	-802.926	-503844.1
Enthalpy	-802.9251	-503843.5
Gibbs	-802.9877	-503882.8
Entropy	131.927	cal/mol.K

Structure TS4	Hartrees	kcal/mol
Energy	-802.925	-503843.5
Enthalpy	-802.924	-503842.9
Gibbs	-802.9842	-503880.6
Entropy	126.539	cal/mol.K

Structure 1	Hartrees	kcal/mol
Energy	-577.5459	-362415.8
Enthalpy	-577.5450	-362415.3
Gibbs	-577.5999	-362449.7
Entropy	115.505	cal/mol.K

Structure 2	Hartrees	kcal/mol
Energy	-577.3363	-362284.3
Enthalpy	-577.3354	-362283.7
Gibbs	-577.3906	-362318.4
Entropy	116.148	cal/mol.K

Structure 3	Hartrees	kcal/mol
Energy	-803.6045	-504269.8

Enthalpy	-803.6035	-504269.3
Gibbs	-803.6668	-504308.9
Entropy	133.071	cal/mol.K

**Structure 4**	**Hartrees**	**kcal/mol**
Energy	-652.747	-409605.3
Enthalpy	-652.7461	-409604.7
Gibbs	-652.8029	-409640.4
Entropy	119.577	cal/mol.K

**Structure 5**	**Hartrees**	**kcal/mol**
Energy	-652.7623	-409614.9
Enthalpy	-652.7613	-409614.3
Gibbs	-652.8189	-409650.4
Entropy	121.116	cal/mol.K

**Structure 6**	**Hartrees**	**kcal/mol**
Energy	-652.1316	-409219.1
Enthalpy	-652.1306	-409218.5
Gibbs	-652.1885	-409254.8
Entropy	121.771	cal/mol.K

**Structure 7**	**Hartrees**	**kcal/mol**
Energy	-803.0517	-503923
Enthalpy	-803.0508	-503922.4
Gibbs	-803.1136	-503961.8
Entropy	132.168	cal/mol.K l

**Structure 8**	**Hartrees**	**kcal/mol**
Energy	-574.2225	-360330.4
Enthalpy	-574.2216	-360329.8
Gibbs	-574.2713	-360361
Entropy	104.707	cal/mol.K

**Hidrogen ion**	**Hartrees**	**kcal/mol**
Energy	-0.163147	-102.3764
Enthalpy	-0.162203	-101.784
Gibbs	-0.174563	-109.54
Entropy	26.014	cal/mol.K

**Water**	**Hartrees**	**kcal/mol**

Energy	-76.37911	-47928.65
Enthalpy	-76.37816	-47928.06
Gibbs	-76.40024	-47941.92
Entropy	46.475	cal/mol.K

**OOH radical**	**Hartrees**	**kcal/mol**
Energy	-150.8354	-94650.74
Enthalpy	-150.8345	-94650.14
Gibbs	-150.8604	-94666.42
Entropy	54.602	cal/mol.K

**Hidrogen radical**	**Hartrees**	**kcal/mol**
Energy	-0.496912	-311.8172
Enthalpy	-0.495968	-311.2249
Gibbs	-0.508982	-319.3913
Entropy	27.392	cal/mol.K

**Acetic acid**	**Hartrees**	**kcal/mol**
Energy	-228.9431	-143664.1
Enthalpy	-228.9422	-143663.5
Gibbs	-228.9746	-143683.8
Entropy	68.195	cal/mol.K

**Oxygen**	**Hartrees**	**kcal/mol**
Energy	-149.5267	-93829.51
Enthalpy	-149.5258	-93828.92
Gibbs	-149.5479	-93842.82
Entropy	4.979	cal/mol.K

**Step TS1**	**Reaction molar standar**	**Activation**
	**kcal/mol**	**kcal/mol**
ΔE^0 = (Structure 4 + OOH radical) – Structure 3<br>E^A = TS1 - Structure 3		
Energy	13.81	32.80
Enthalpy	14.40	32.80
Gibbs	2.15	32.92
Entropy	41.11	-0.42
PV	0.59	0.00

**Step TS2**	**Reaction molar standar**	**Activation**
	**kcal/mol**	**kcal/mol**
ΔE^0 = Structure 5 – Structure 4<br>E^A = TS2 - Structure 4		
Energy	-9.56	49.03
Enthalpy	-9.56	49.03
Gibbs	-10.01	49.36
Entropy	1.54	-1.10
pv	0.00	0.00

**Step TS3**	**Reaction molar standar**	**Activation**
	**kcal/mol**	**kcal/mol**
ΔE^0 = Structure 7 – (Structure 6 + OOH rad)<br>E^A = TS3 - (Structure 6 + OOH rad)		
Energy	-53.17	25.71
Enthalpy	-53.76	25.12
Gibbs	-40.58	38.37
Entropy	-44.21	-44.45
pv	-0.59	-0.59

**Step TS4**	**Reaction molar standar**	**Activation**
	**kcal/mol**	**kcal/mol**
ΔE^0 = (Structure 8 + Acetic acid) – Structure 7<br>E^A = TS4 – Structure 7		
Energy	-71.50	79.52
Enthalpy	-70.91	79.52
Gibbs	-83.06	81.20
Entropy	40.73	-5.63
pv	0.59	0.00
