# Supplementary material for: On the Reaction Mechanism of the 3,4-Dimethoxybenzaldehyde Formation from 1-(3′,4′-Dimethoxyphenyl)Propene
Source: Molecules. 2018 Feb 14;23(2):412. doi: 10.3390/molecules23020412 (PMC6017041; doi:10.3390/molecules23020412)
Supplement: Supplementary file 1 [file molecules-23-00412-s001.zip › Supplementary material 2..pdf]

Table S2: Frequencies of Transition states

TS1

Mode #	Freq	Infrared
1	-842.67	1046.7297

TS2

Mode #	Freq	Infrared
1	-2197.37	2162.0733

TS3

Mode #	Freq	Infrared
1	-433.46	995.5108

TS4

Mode #	Freq	Infrared
1	-1151.23	1221.3764
