# Supplementary material for: On the Reaction Mechanism of the 3,4-Dimethoxybenzaldehyde Formation from 1-(3′,4′-Dimethoxyphenyl)Propene
Source: Molecules. 2018 Feb 14;23(2):412. doi: 10.3390/molecules23020412 (PMC6017041; doi:10.3390/molecules23020412)
Supplement: Supplementary file 1 [file molecules-23-00412-s001.zip › Supplementary material 3..pdf]

Table S3: Kinetic Parameters

TS1		
# Total Energy along IRC		
# X-Axis: Intrinsic Reaction Coordinate		
# Y-Axis: Total Energy (kcal/mol)		
Points	X	Y
1	2.86	-504414.18
2	2.72	-504413.36
3	2.58	-504412.54
4	2.43	-504411.74
5	2.29	-504410.94
6	2.15	-504410.17
7	2.00	-504409.40
8	1.86	-504408.66
9	1.72	-504407.94
10	1.57	-504407.24
11	1.43	-504406.57
12	1.29	-504405.93
13	1.14	-504405.31
14	1.00	-504404.73
15	0.85	-504404.19
16	0.71	-504403.68
17	0.57	-504403.21
18	0.42	-504402.79
19	0.28	-504402.39
20	0.14	-504402.12
21	0.00	-504401.99
22	-0.14	-504402.22
23	-0.29	-504403.06
24	-0.43	-504404.61
25	-0.57	-504406.71
26	-0.72	-504408.90
27	-0.86	-504410.65
28	-1.00	-504412.04
29	-1.14	-504413.29
30	-1.28	-504414.45
31	-1.43	-504415.54
32	-1.57	-504416.57
33	-1.71	-504417.53
34	-1.86	-504418.43
35	-2.00	-504419.28
36	-2.14	-504420.07
37	-2.29	-504420.80
38	-2.43	-504421.48

39	-2.57	-504422.10
40	-2.72	-504422.69
41	-2.86	-504423.23

**TS1 Reaction Force**		
Point	Reaction coordinate	Reaction Force
1	-2.86	-5.75
2	-2.72	-5.68
3	-2.57	-5.62
4	-2.43	-5.51
5	-2.29	-5.42
6	-2.14	-5.31
7	-2.00	-5.16
8	-1.86	-5.05
9	-1.71	-4.84
10	-1.57	-4.67
11	-1.43	-4.48
12	-1.28	-4.28
13	-1.14	-4.07
14	-1.00	-3.90
15	-0.86	-3.64
16	-0.72	-3.29
17	-0.57	-2.90
18	-0.43	-2.81
19	-0.29	-1.88
20	-0.14	-0.89
21	0.00	1.61
22	0.14	5.98
23	0.28	10.83
24	0.42	14.63
25	0.57	15.25
26	0.71	12.20
27	0.85	9.73
28	1.00	8.69
29	1.14	8.09
30	1.29	7.56
31	1.43	7.14
32	1.57	6.68
33	1.72	6.33
34	1.86	5.88
35	2.00	5.49
36	2.15	5.10
37	2.29	4.72
38	2.43	4.37

39	2.58	4.11
40	2.72	3.78

**TS1 Force constant**		
Point	Reaction coordinate	Force constant
1	-2.86	-0.48
2	-2.72	-0.42
3	-2.57	-0.76
4	-2.43	-0.62
5	-2.29	-0.77
6	-2.14	-1.01
7	-2.00	-0.80
8	-1.86	-1.44
9	-1.71	-1.20
10	-1.57	-1.34
11	-1.43	-1.39
12	-1.28	-1.49
13	-1.14	-1.17
14	-1.00	-1.82
15	-0.86	-2.49
16	-0.72	-2.74
17	-0.57	-0.65
18	-0.43	-6.48
19	-0.29	-6.86
20	-0.14	-17.62
21	0.00	-31.22
22	0.14	-34.50
23	0.28	-26.51
24	0.42	-4.32
25	0.57	21.26
26	0.71	17.24
27	0.85	7.20
28	1.00	4.18
29	1.14	3.74
30	1.29	2.87
31	1.43	3.21
32	1.57	2.46
33	1.72	3.12
34	1.86	2.72
35	2.00	2.73
36	2.15	2.66
37	2.29	2.47
38	2.43	1.81
39	2.58	2.30

**TS2**		
**# Total Energy along IRC**		
**# X-Axis: Intrinsic Reaction Coordinate**		
**# Y-Axis: Total Energy (kcal/mol)**		
Points	X	Y
1	2.09	-409752.52
2	1.99	-409751.51
3	1.88	-409750.39
4	1.78	-409749.12
5	1.67	-409747.70
6	1.57	-409746.12
7	1.46	-409744.37
8	1.36	-409742.44
9	1.26	-409740.32
10	1.15	-409737.98
11	1.05	-409735.42
12	0.94	-409732.62
13	0.84	-409729.56
14	0.73	-409726.24
15	0.63	-409722.69
16	0.52	-409718.98
17	0.42	-409715.31
18	0.31	-409711.95
19	0.21	-409709.22
20	0.10	-409707.41
21	0.00	-409706.78
22	-0.10	-409707.39
23	-0.21	-409709.12
24	-0.31	-409711.81
25	-0.42	-409715.33
26	-0.52	-409719.53
27	-0.63	-409724.19
28	-0.73	-409729.09
29	-0.84	-409733.97
30	-0.94	-409738.63
31	-1.05	-409742.94
32	-1.15	-409746.85
33	-1.26	-409750.33
34	-1.36	-409753.40
35	-1.47	-409756.04
36	-1.57	-409758.27
37	-1.67	-409760.11
38	-1.78	-409761.59
39	-1.88	-409762.74
40	-1.99	-409763.64
41	-2.09	-409764.34

TS2 Reaction Force		
Point	Reaction coordinate	Reaction Force
1	-2.09	-9.64
2	-1.99	-10.78
3	-1.88	-12.17
4	-1.78	-13.58
5	-1.67	-15.09
6	-1.57	-16.70
7	-1.47	-18.44
8	-1.36	-20.30
9	-1.26	-22.32
10	-1.15	-24.50
11	-1.05	-26.80
12	-0.94	-29.21
13	-0.84	-31.67
14	-0.73	-33.98
15	-0.63	-35.46
16	-0.52	-35.05
17	-0.42	-32.04
18	-0.31	-26.16
19	-0.21	-17.30
20	-0.10	-6.02
21	0.00	5.83
22	0.10	16.55
23	0.21	25.74
24	0.31	33.63
25	0.42	40.09
26	0.52	44.63
27	0.63	46.82
28	0.73	46.65
29	0.84	44.57
30	0.94	41.24
31	1.05	37.33
32	1.15	33.32
33	1.26	29.32
34	1.36	25.27
35	1.46	21.32
36	1.57	17.56
37	1.67	14.09
38	1.78	11.05
39	1.88	8.59
40	1.99	6.84

TS2 Force constant		
Point	Reaction coordinate	Force constant
1	-2.09	10.94
2	-1.99	13.32
3	-1.88	13.47
4	-1.78	14.39
5	-1.67	15.40
6	-1.57	16.62
7	-1.47	17.76
8	-1.36	19.32
9	-1.26	20.88
10	-1.15	21.96
11	-1.05	23.07
12	-0.94	23.49
13	-0.84	22.06
14	-0.73	14.14
15	-0.63	-3.96
16	-0.52	-28.71
17	-0.42	-56.16
18	-0.31	-84.71
19	-0.21	-107.79
20	-0.10	-113.22
21	0.00	-102.37
22	0.10	-87.87
23	0.21	-75.38
24	0.31	-61.72
25	0.42	-43.42
26	0.52	-20.94
27	0.63	1.63
28	0.73	19.88
29	0.84	31.93
30	0.94	37.37
31	1.05	38.30
32	1.15	38.21
33	1.26	38.71
34	1.36	37.76
35	1.46	35.94
36	1.57	33.16
37	1.67	29.10
38	1.78	23.51
39	1.88	16.80

TS3		
**# Total Energy along IRC**		
**# X-Axis: Intrinsic Reaction Coordinate**		
**# Y-Axis: Total Energy (kcal/mol)**		
Points	X	Y
1	6.84	-504034.00
2	6.49	-504033.54
3	6.15	-504032.99
4	5.80	-504032.37
5	5.45	-504031.67
6	5.10	-504030.89
7	4.76	-504030.03
8	4.41	-504029.08
9	4.06	-504028.03
10	3.71	-504026.84
11	3.36	-504025.46
12	3.02	-504023.81
13	2.67	-504021.78
14	2.34	-504019.30
15	2.00	-504015.87
16	1.66	-504010.90
17	1.39	-504006.92
18	1.05	-504005.23
19	0.70	-504004.06
20	0.35	-504003.30
21	0.00	-504003.03
22	-0.35	-504003.29
23	-0.70	-504003.94
24	-1.03	-504004.58
25	-1.30	-504004.91
26	-1.63	-504005.21
27	-1.98	-504005.50
28	-2.32	-504005.76
29	-2.66	-504005.98
30	-2.98	-504006.13
31	-3.28	-504006.22
32	-3.61	-504006.30
33	-3.95	-504006.37
34	-4.29	-504006.44

TS3 Reaction Force		
Point	Reaction coordinate	Reaction Force
1	-4.29	-1.38
2	-3.95	-1.60
3	-3.61	-1.90
4	-3.28	-2.37
5	-2.98	-2.43
6	-2.66	-2.52
7	-2.32	-2.74
8	-1.98	-3.04
9	-1.63	-3.59
10	-1.30	-5.22
11	-1.03	-4.90
12	-0.70	-5.82
13	-0.35	-7.13
14	0.00	-9.84
15	0.35	-14.26
16	0.70	-11.43
17	1.05	-4.89
18	1.39	-4.35
19	1.66	-2.23
20	2.00	-0.80
21	2.34	0.77
22	2.67	1.87
23	3.02	1.85
24	3.36	0.96
25	3.71	0.86
26	4.06	0.82
27	4.41	0.75
28	4.76	0.62
29	5.10	0.43
30	5.45	0.27
31	5.80	0.23
32	6.15	0.21
33	6.49	0.20

TS3 Force constant		
Point	Reaction coordinate	Force constant
1	-4.29	0.64
2	-3.95	0.89
3	-3.61	1.43
4	-3.28	0.20
5	-2.98	0.30
6	-2.66	0.62
7	-2.32	0.87
8	-1.98	1.60
9	-1.63	4.92
10	-1.30	-1.21
11	-1.03	2.74
12	-0.70	3.76
13	-0.35	7.77
14	0.00	12.68
15	0.35	-8.10
16	0.70	-18.79
17	1.05	-1.57
18	1.39	-7.85
19	1.66	-4.20
20	2.00	-4.69
21	2.34	-3.26
22	2.67	0.05
23	3.02	2.58
24	3.36	0.27
25	3.71	0.14
26	4.06	0.19
27	4.41	0.37
28	4.76	0.54
29	5.10	0.48
30	5.45	0.11
31	5.80	0.04
32	6.15	0.00

TS4		
**# Total Energy along IRC**		
**# X-Axis: Intrinsic Reaction Coordinate**		
**# Y-Axis: Total Energy (kcal/mol)**		
Points	X	Y
1	6.11	-504082.90
2	5.80	-504082.49
3	5.50	-504082.03
4	5.20	-504081.50
5	4.89	-504080.87
6	4.59	-504080.14
7	4.28	-504079.25
8	3.98	-504078.18
9	3.67	-504076.88
10	3.37	-504075.31
11	3.06	-504073.46
12	2.76	-504071.24
13	2.45	-504068.51
14	2.15	-504065.04
15	1.84	-504060.74
16	1.53	-504055.68
17	1.23	-504050.07
18	0.92	-504044.36
19	0.61	-504039.27
20	0.30	-504035.70
21	0.00	-504034.47
22	-0.31	-504036.36
23	-0.61	-504041.45
24	-0.92	-504048.87
25	-1.23	-504057.49
26	-1.54	-504066.56
27	-1.84	-504075.71
28	-2.15	-504084.73
29	-2.46	-504093.46
30	-2.77	-504101.80
31	-3.07	-504109.65
32	-3.38	-504116.90
33	-3.69	-504123.39
34	-4.00	-504129.03
35	-4.30	-504133.70
36	-4.61	-504137.56
37	-4.92	-504140.73
38	-5.22	-504143.37
39	-5.53	-504145.60
40	-5.83	-504147.46
41	-6.14	-504149.05

TS4 Reaction Force		
Point	Reaction coordinate	Reaction Force
1	-6.14	-1.33
2	-5.83	-1.50
3	-5.53	-1.76
4	-5.22	-2.03
5	-4.92	-2.41
6	-4.61	-2.88
7	-4.30	-3.48
8	-4.00	-4.23
9	-3.69	-5.10
10	-3.38	-6.03
11	-3.07	-7.21
12	-2.77	-8.91
13	-2.46	-11.27
14	-2.15	-13.98
15	-1.84	-16.46
16	-1.54	-18.25
17	-1.23	-18.60
18	-0.92	-16.54
19	-0.61	-11.62
20	-0.31	-4.03
21	0.00	6.24
22	0.30	16.58
23	0.61	24.11
24	0.92	28.04
25	1.23	29.52
26	1.53	29.77
27	1.84	29.36
28	2.15	28.63
29	2.45	27.45
30	2.76	25.83
31	3.06	23.76
32	3.37	21.18
33	3.67	18.46
34	3.98	15.30
35	4.28	12.64
36	4.59	10.42
37	4.89	8.69
38	5.20	7.30
39	5.50	6.18
40	5.80	5.22

TS4 Force constant		
Point	Reaction coordinate	Force constant
1	-6.14	0.57
2	-5.83	0.83
3	-5.53	0.90
4	-5.22	1.22
5	-4.92	1.55
6	-4.61	1.95
7	-4.30	2.45
8	-4.00	2.84
9	-3.69	3.01
10	-3.38	3.82
11	-3.07	5.53
12	-2.77	7.71
13	-2.46	8.80
14	-2.15	8.07
15	-1.84	5.81
16	-1.54	1.14
17	-1.23	-6.70
18	-0.92	-16.00
19	-0.61	-24.73
20	-0.31	-33.48
21	0.00	-34.02
22	0.30	-24.55
23	0.61	-12.78
24	0.92	-4.82
25	1.23	-0.82
26	1.53	1.34
27	1.84	2.39
28	2.15	3.85
29	2.45	5.33
30	2.76	6.83
31	3.06	8.44
32	3.37	8.90
33	3.67	10.31
34	3.98	8.72
35	4.28	7.30
36	4.59	5.68
37	4.89	4.55
38	5.20	3.68
39	5.50	3.19
